# PGK1 enhances productive bovine herpesvirus 1 infection by stimulating β-catenin-dependent transcription

**DOI:** 10.1186/s13567-025-01480-5

**Published:** 2025-03-07

**Authors:** Xuan Li, Wenyuan Gu, Shitao Li, Filomena Fiorito, Xiuyan Ding, Liqian Zhu

**Affiliations:** 1 Key Laboratory of Microbial Diversity Research and Application of Hebei Province, School of Life Sciences, Hebei University, Baoding, 071002 China; 2 Center for Animal Diseases Control and Prevention of Hebei Province, Shijiazhuang, 050035 China; 3 Department of Microbiology and Immunology, Tulane University, New Orleans, LA 70118 USA; 4 Department of Veterinary Medicine and Animal Production, University of Naples Federico II, 80137 Naples, Italy

**Keywords:** BoHV-1, PGK1, β-Catenin signalling, mitochondria

## Abstract

Bovine herpesvirus 1 (BoHV-1) productive infection stimulates β-catenin-dependent transcription to facilitate virus replication. Phosphoglycerate kinase 1 (PGK1), which catalyses the initial step of ATP production during glycolysis, also has a mitochondrial form that is implicated in tissue injury across various diseases. However, the relationship between BoHV-1 replication and the PGK1 signalling pathway is not yet fully understood. In this study, we discovered that PGK1 signalling significantly influences BoHV-1 replication, with the virus infection leading to a marked increase in the accumulation of PGK1 proteins in mitochondria. Overexpression of β-catenin reduces PGK1 steady-state protein levels while overexpressing PGK1 boosts β-catenin protein expression—a phenomenon that reverses upon virus infection. Importantly, consistent with PGK1’s vital role in virus replication, PGK1 stimulates β-catenin-dependent transcriptional activity, partly by promoting the nuclear accumulation of transcriptionally active β-catenin and phospho-β-catenin (S552) in virus-infected cells. In summary, our findings suggest for the first time that PGK1 signalling may be involved in BoHV-1 replication and contribute to virus pathogenicity.

## Introduction

Bovine herpesvirus 1 (BoHV-1) is prevalent in cattle globally. Infections caused by BoHV-1 infection can lead to various diseases ranging from infectious bovine rhinotracheitis (IBR) to infectious pustular vulvovaginitis, abortion, and systemic infection in neonatal animals [1, 2]. The infection typically starts at the mucosa, resulting in generally non-life-threatening symptoms. However, as the virus replicates, it can cause erosion of the respiratory tract and suppress immune responses. This suppression may contribute to facilitating the invasion of other pathogens, such as bovine respiratory syncytial virus (BRSV), parainfluenza-3 virus (PI3V), bovine coronaviruses, *Mannheimia haemolytica*, *Pasteurella multocida*, *Histophilus somni*, and *Mycoplasma spp*. Such invasions may lead to life-threatening pneumonia, known as bovine respiratory disease complex (BRDC), which is the most significant disease affecting cattle [3, 4] and poses a substantial economic burden on the cattle industry worldwide [1, 5]. Additionally, BoHV-1 also impacts endangered bovine species such as mithun and yak [6, 7].

Adenosine 5ʹ-triphosphate (ATP), generated by glucose metabolism within host cells, is essential for virus replication. Glycolysis is a critical energy-producing pathway that converts glucose into pyruvate, producing ATP and NADPH. Numerous studies have suggested that virus infections can reprogram the host’s metabolism to enhance their replication cycles [8]. For instance, the glycolytic metabolism is upregulated by Senecavirus A [8], SARS-CoV-2 [9], and dengue virus [10] to facilitate viral replication. However, the relationship between glycolysis and BoHV-1 infection is still not fully understood. Although the activation of the glycolysis metabolism has been reported in herpes simplex virus 1 (HSV-1) [11, 12], which is genetically close to BoHV-1, further investigation is needed.

During glycolysis, glucose is metabolised into pyruvate. Under anaerobic conditions, the expression levels of key glycolytic enzymes—HK2, PFKM, PKM, and PGK1—increase, and pyruvate is metabolised by lactate dehydrogenase (LDH) to form large amounts of lactate [8, 13]. PGK1 catalyses the initial ATP-generating steps in the glycolytic pathway through substrate-level phosphorylation of ADP and DPG in the cytosol [14, 15]. Research indicates that PGK1 is crucial for balancing energy production with biosynthesis and redox status [16]. Additionally, PGK1 plays a role in various biological activities, including angiogenesis, autophagy, DNA replication and DNA damage repair, tumour growth, as well as cell proliferation and metastasis in many types of human tumour cells [17, 18].

The virus takes over cellular metabolites and energy to complete replication cycles, which may affect the host metabolism and result in disease development. We have previously reported that BoHV-1 productive infection at later stages leads to depletion of ATP levels in cell cultures and mitochondrial dysfunction [19], while the mechanism regarding virus infection-induced ATP depletion remains to be determined. With this in mind, we explored whether BoHV-1 productive infection coordinates the PGK1 signalling pathway to facilitate viral replication cycles.

Previous studies have indicated that β-catenin signalling stimulates BoHV-1 productive infection in cell cultures [20]. It also plays a role in regulating glycolysis through various mechanisms [21–23]. For instance, β-catenin signalling positively influences the expression of the glycolytic target genes GLUT1, HK2, PKM2, and LDHA [21, 22]. Additionally, β-catenin may act as a downstream target of PGK1 [24]. A recent study found that disrupting the interaction between HSP90 and PGK1 leads to stabilisation of β-catenin expression [25]. Together, these findings suggest a potential interplay between β-catenin and PGK1.

Our study discovered that PGK1 is crucial for productive infection by BoHV-1. PGK1 enhances β-catenin-dependent transcription in cells infected with the virus, revealing a new mechanism through which PGK1 aids in virus replication. Additionally, BoHV-1 productive infection increases the accumulation of PGK1 proteins in the mitochondria. This finding adds a new dimension to our understanding of the virus’s pathogenicity, as mitochondrial PGK1 can worsen tissue damage in various diseases. Consequently, PGK1 may serve as a promising therapeutic target for developing new strategies to target BoHV-1 infection.

## Materials and methods

### Cell cultures

MDBK cells were obtained from the Chinese Model Culture Preservation Center in Shanghai, China. Professor Dongli Pan from Zhejiang University kindly provided Neuro-2A cells. These cells were routinely maintained and passaged in DMEM supplemented with 10% fetal bovine serum (FBS).

### Viruses and plasmids

The BoHV-1 strain NJ-16-1, isolated from bovine semen samples [26], was propagated in MDBK cells. Aliquots of virus stocks were stored at −70 °C until needed. The Super 8 × TOPFlash, which contained a promoter stimulated by β-catenin, was a gift from Randall Moon (Addgene plasmid # 12456) [27]. The human β-catenin expression construct (S33Y), which expressed a Flag-tagged β-catenin protein, was a gift from Bert Vogelstein (Addgeneplasmid#16519) [28]. Additionally, the plasmid pcDNA3.1-PGK1 was kindly provided by Professor Xinping Chen at Lanzhou University [29].

### Antibodies and reagents

The following antibodies were used in this study: PGK1 rabbit pAb (cat# A14039), GAPDH rabbit pAb (cat# AC027), β-Actin rabbit pAb (cat# AC026), and β-Tubulin Rabbit pAb (cat# AC015). These antibodies were all ordered from Abclonal Technology (Woburn, MA, USA). β-catenin rabbit mAb (cat# 8480S), p-β-catenin (S552) Antibody (cat# 9566S), COXIV rabbit pAb (cat# 4844S), HRP conjugated goat anti-mouse IgG (cat# 7076), and HRP labelled goat anti-rabbit IgG (cat# 7074) were purchased from Cell Signaling Technology (Danvers, MA, USA). LaminA/C mouse mAb (cat# sc-376248) was provided by Santa Cruz Biotechnology (Dallas, TX, USA). Alexa Fluor 488®-conjugated goat anti-rabbit IgG (H+L) (cat# A-11008) was provided by Invitrogen Life Technologies (Waltham, MA, USA). β-catenin-specific inhibitor iCRT14 (cat# HY-16665) and PGK1-specific inhibitor NG52 (cat# HY-15154) were ordered from MedChemExpress (Monmouth Junction, NJ, USA). Finally, Lipofectamine 3000 (Invitrogen), and PGK1-specific siRNA were provided by Genepharma (Shanghai, China).

### Western blotting analysis

Cell lysates were prepared from whole cell extracts or specific cellular fractions, including mitochondria, nucleus, cell membrane, and cytoplasm, using RIPA lysis buffer (1× PBS, 1% NP-40, 0.5% sodium deoxycholate, 0.1% SDS) supplemented with a protease inhibitor cocktail. The samples were then boiled in Laemmli sample buffer for 5 min, subjected to separation on SDS-PAGE (8% or 10%), and transferred to polyvinylidene fluoride (PVDF) membranes. Immuno-reactive bands were developed using Clarity Western ECL Substrate (Bio-Rad, cat# 1705061).

The band intensity was quantitatively analysed for the designated studies with the free software program Image J. Significance was assessed using a Student’s *t*-test with GraphPad Prism software (v8.0). *p* values of less than 0.05 (*p < 0.05) were considered significant for all calculations.

### Immunofluorescence assay

MDBK cells in eight-well chamber slides (Nunc Inc., IL, USA) were either mock-infected or infected with BoHV-1 (MOI = 1). After 24 h (h) of infection, the cells were fixed with 4% paraformaldehyde in PBS for 10 min at room temperature. They were then permeabilised with 0.25% Triton X-100 in PBS for 10 min at room temperature and blocked with 1% BSA in PBST for one hour. The cells were incubated overnight at 4 °C with the indicated antibody in 1% BSA in PBST. After three washings, the cells were incubated with a secondary antibody labelled with distinct fluorescent dyes for one hour in the dark at room temperature. After three washings with PBS, the nuclei were stained with DAPI (4′,6-diamidino-2-phenylindole). Finally, slides were covered with coverslips using an antifade mounting medium (Electron Microscopy Sciences, cat# 50-247-04). Images were captured using a confocal microscope (Zeiss).

### Immunoprecipitation assay

Cellular lysates were obtained from either virus-infected cells or mock-infected cells. These lysates were clarified by centrifugation at 16 000 rpm for 10 min. They were then incubated with Dynabeads protein A (Life Technologies, cat# 10001D), which had been precoated with antibodies against either PGK1 pA or β-catenin. This incubation took place for one hour at room temperature with rotation. An isotype IgG was used as a control.

Following overnight incubation at 4 °C with rotation, the Dynabeads were collected using a magnet (DynaMag) (Life Technologies, cat# 12321D). After three washings with PBS, the beads were boiled in an SDS-loading buffer and subjected to western blot analysis.

### RNA isolation and quantification of mRNA by qRT-PCR

Total RNA was extracted from cells subjected to virus infection or plasmid/siRNA transfection using TRIzol LS reagent (Ambion, cat#10296010), following the manufacturer’s instructions. Freshly prepared total RNA (1 μg) served as the template for synthesising first-strand cDNA with commercial random hexamer primers, using the Thermoscript™ RT-PCR system Kit (Invitrogen, cat#11146-024), following the manufacturer's instructions. The resulting cDNA products were then utilised as real-time quantitative PCR templates to measure the indicated genes' mRNA levels with gene-specific primers. For these studies, the primers were used as follows: mouse-derived PGK-1 (forward primer 5′-TTGTGCATTGTAGAGGGCGT-3′ and reverse primer: 5′-TGACGAAGCTAACCAGAGGC-3′), bovine-derived PGK-1 (forward primer 5′-TCTCTGCCGCTGTCTCATCC-3′ and reverse primer: 5′-ACTCTCATGACGACCCGCTT-3′), gC (forward primer 5′-ACTATATTTTCCCTTCGCCCG-3′ and reverse primer: 5′-TGTGACTTGGTGCCCATG-3′). GAPDH (forward primer: 5′-CCATGGAGAAGGCTGGGG-3′ and reverse primer: 5′-AAGTTGTCATGGATGACC-3′), bICP27 (forward primer 5′-AAACCTGGTAGACGCACTGG-3′ and reverse primer 5′-ACGATAGGGTCTTTGGTGCG-3′), and VP16 (forward primer 5′-GCGCTGGGCTTCCTGAATTA-3′ and reverse primer 5′-TCTGGAGTCGTCCCGTAGTT-3′) [30, 31].

Glyceraldehyde-3-phosphate dehydrogenase (GAPDH) mRNA was used as an internal control for the analysis. Real-time PCR was conducted using the ABI 7500 fast real-time system (Applied Biosystems, CA, USA). The expression levels of the tested genes were normalised to that of either the GAPDH or β-Actin gene. The relative mRNA level of each gene was calculated using the method (2^−ΔΔCT^), comparing it to the control.

### DNA purification and quantification of viral DNA by qPCR

MDBK cells with either PGK1 knockdown or inhibited PGK1 activity using the chemical inhibitor NG52 were infected with BoHV-1. Cellular DNA was purified using a commercial viral DNA purification kit (Tiangen, cat# DP-348), following the manufacturer’s instructions. The freshly prepared DNA served as a template for real-time qPCR to measure viral DNA levels using gC-specific primers, as described above.

GAPDH analysis was performed as an internal control. Real-time PCR was conducted using the LightCycler 96 fast real-time system (Roche, CHE). The levels of the viral genome represented by gC were normalised to those of the GAPDH gene. The relative levels of the viral genome were calculated using the 2^−ΔΔCT^ method compared to the control.

### siRNA transfection assay

MDBK cells in six-well plates were transfected with either scrambled siRNA or three 200 pmol PGK1-specific siRNAs provided by Genepharma (Shanghai, China). At 36 or 48 h post-transfection, cell lysates were prepared using the described cell lysis buffer. These lysates were then analysed using western blot to detect the protein levels of PGK1. Additionally, some cells were infected with BoHV-1 (MOI = 0.1) for 24 h. The progeny viruses were detected in MDBK cells, with results expressed as TCID_50_/mL and calculated using the Reed-Muench formula.

### NG52 treatment of MDBK cells during virus infection

MDBK confluent cells in 24-well plates were infected with BoHV-1 (MOI = 0.1). The cells were treated with the chemical NG52 (MCE, cat# HY-15154) at a specified concentration for 1 h at 37 °C. Following this treatment, the cells were washed three times with PBS, and a fresh medium containing NG52 at the designated concentrations was added to each well. After 12 and 24 h of infection, viral yields were titrated in MDBK cells. Cell cultures treated with DMSO served as a control. The results were expressed as TCID_50_/mL and calculated using the Reed-Muench formula.

### Dual-luciferase reporter assay

Neuro-2A or MDBK cells were co-transfected with the Super 8 × TOPFlash plasmid and a plasmid encoding Renilla luciferase under the control of a minimal herpesvirus promoter (Promega). This was done with or without adding the PGK1 plasmid using Lipofectamine 3000 Transfection Reagent (Invitrogen). The cells were harvested at the designated times after infection, and protein extracts were subjected to a dual-luciferase assay using a commercially available kit (E1910; Promega) in accordance with the manufacturer’s instructions. Luminescence was measured with a GloMax 20/20 luminometer (E5331; Promega).

### Statistical analysis

All data analyses were conducted using Prism software 8.3 and IBM SPSS Statistics, version 25. The experimental data are presented as mean value ± standard deviation (SD). A one-way analysis of variance (ANOVA) was used to compare multiple groups. *p*-values less than 0.05 were considered statistically significant.

## Results

### BoHV-1 productive infection decreases PGK1 protein expression

**Figure 1:**
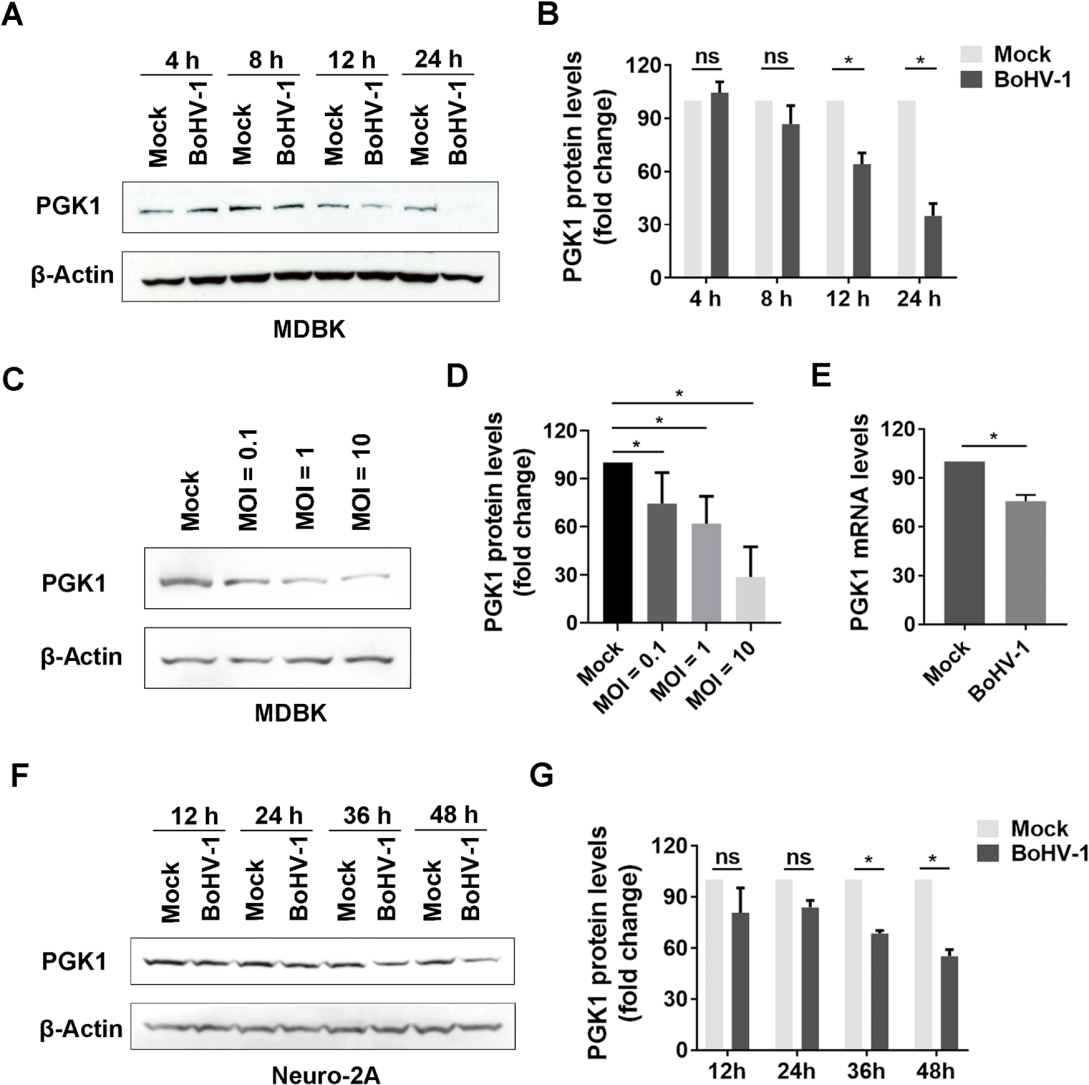
**BoHV-1 productive infection decreases PGK1 protein expression in MDBK cells.** **A** MDBK cells in 60 mm dishes were either mock infected or infected with BoHV-1 at an MOI of 1. After infection for indicated time lengths, cell lysates were prepared and analysed by western blotting to detect PGK1 protein levels, using β-Actin as a loading control and subsequent quantitative analysis. **C** MDBK cells were either mock infected or infected with BoHV-1 at an MOI of 0.1, 1, and 10, respectively. After infection for 24 h, cell lysates were prepared and subjected to western blotting to detect the PGK1 protein, respectively. **B**, **D** and **G** The band intensity was analysed using free software image J. In detail, the band intensity of PGK1 protein was initially normalised to β-Actin, and the fold change after infection was calculated by comparison to that of mock-infected control, which was arbitrarily set as 100%. **E** MDBK cells in 6-well plates were either mock infected or infected with BoHV-1 at an MOI of 1. At 24 hpi, total RNA was purified, and the mRNA levels of PGK1 were examined with qRT-PCR. **F** Neuro-2A cells in 60 mm dishes were either mock infected or infected with BoHV-1 at an MOI of 1. After infection for the specified periods, cell lysates were prepared and analysed by western blotting to detect PGK1 protein levels. The data shown are means of three independent experiments with error bars indicating standard deviations. Significance was assessed with a Student’s *t*-test (*p < 0.05, ns: not significant).

To investigate the role of PGK1 signalling during in vitro infection, we first examined whether BoHV-1 infection affects PGK1 protein expression in MDBK cells. MDBK cells were either mock-infected or infected with BoHV-1 (MOI = 1) for 4, 8, 12, and 24 h. We observed lower levels of PGK1 protein in MDBK cells infected with BoHV-1 at 12 and 24 h after infection (Figure 1A). Specifically, PGK1 protein levels decreased to approximately 64.11% and 35.01% at 12 and 24 hpi, respectively, compared to the control group (Figure 1B). Additionally, when MDBK cells were infected with increasing MOI values ranging from 0.1 to 10 for 24 h, we found a gradual decrease in PGK1 protein levels that correlated with the rising MOI (Figures 1C and D). This further supports the conclusion that the observed reduction in PGK1 proteins is associated with virus replication. As previously reported [30], PGK1 mRNA levels also significantly decreased 24 h after infection (Figure 1E), which aligns with the reduction in protein levels. Therefore, both PGK1 protein and mRNA levels were diminished in response to virus infection.

BoHV-1 can infect the mouse neuroblastoma cell line (Neuro-2A), although this occurs with lower efficiency [32–34]. In this study, Neuro-2A cells were used to investigate the effect of BoHV-1 infection on the expression of PGK1 protein in neuronal cells. The results showed that PGK1 protein levels decreased in virus-infected Neuro-2A cells at 36 and 48 hpi (Figure 1F). Specifically, compared to the control, PGK1 protein levels declined to approximately 68.48% and 55.10% after infection for 36 h and 48 h, respectively (Figure 1G). These findings suggest that BoHV-1 productive infection reduces PGK1 protein expression across different cell types, indicating that this effect is independent of cell type.

### BoHV-1 infection re-localises PGK1 proteins

A previous report indicated that partial PGK1 proteins are located in mitochondria [35]. We aimed to determine whether virus infection affects the subcellular localisation of PGK1, particularly within mitochondria. MDBK cells were stained with Mito-Tracker Deep Red to visualise the mitochondrial network, and an immunofluorescence assay (IFA) was performed using PGK1-specific antibody.

**Figure 2:**
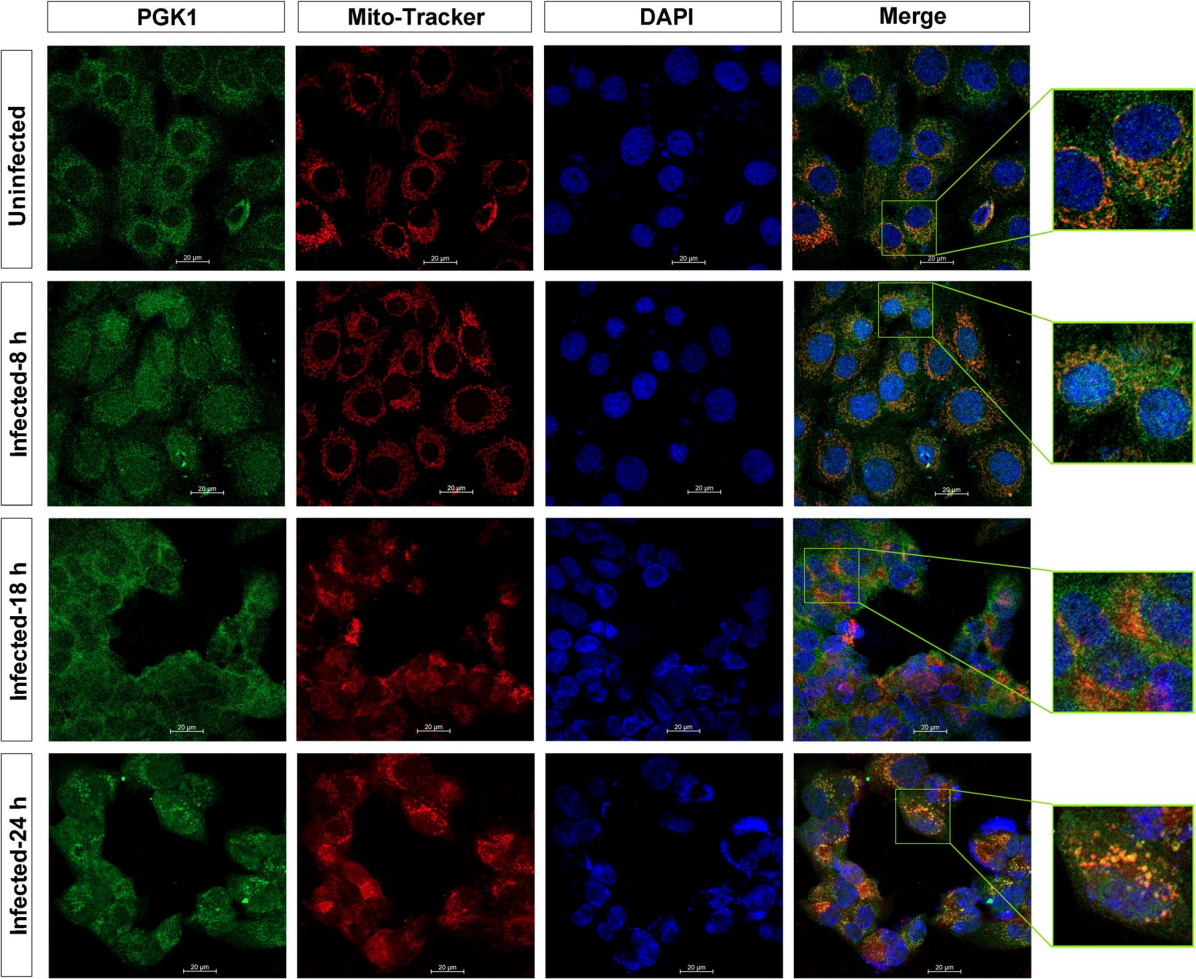
**BoHV-1 infection leads to re-localisation of PGK1 proteins.** MDBK cells were either mock infected or infected with BoHV-1 at an MOI of 1. After infection for 8, 16, and 24 h, mitochondria were labelled with Mito-Tracker Deep Red FM (red) and stained with antibodies against PGK1 (green). Nuclei were stained with DAPI (blue). Then, images were captured using confocal microscopy. Bars = 20 μm.

As shown in Figure 2, PGK1 proteins and mitochondrial networks were evenly distributed in the cytoplasm of both mock-infected cells and virus-infected cells at early stages (8 hpi). However, the mitochondrial networks tended to form aggregates with irregular profiles following virus infection, particularly at later stages (18 and 24 hpi). Notably, partial PGK1 proteins were detected within mitochondrial networks in infected and non-infected cells.

We also found that a subset of PGK1, marked by immunostaining, was prominently present in the aggregated mitochondrial networks of virus-infected cells at 24 h. Significantly, viral infection reduced the steady-state expression of PGK1 protein (Figure 1A). Consequently, a longer exposure time was required during confocal microscopy to detect PGK1 protein levels in the virus-infected cells compared to the uninfected control. Therefore, we caution that direct comparisons of PGK1 levels between these IFA images may not be appropriate.

**Figure 3:**
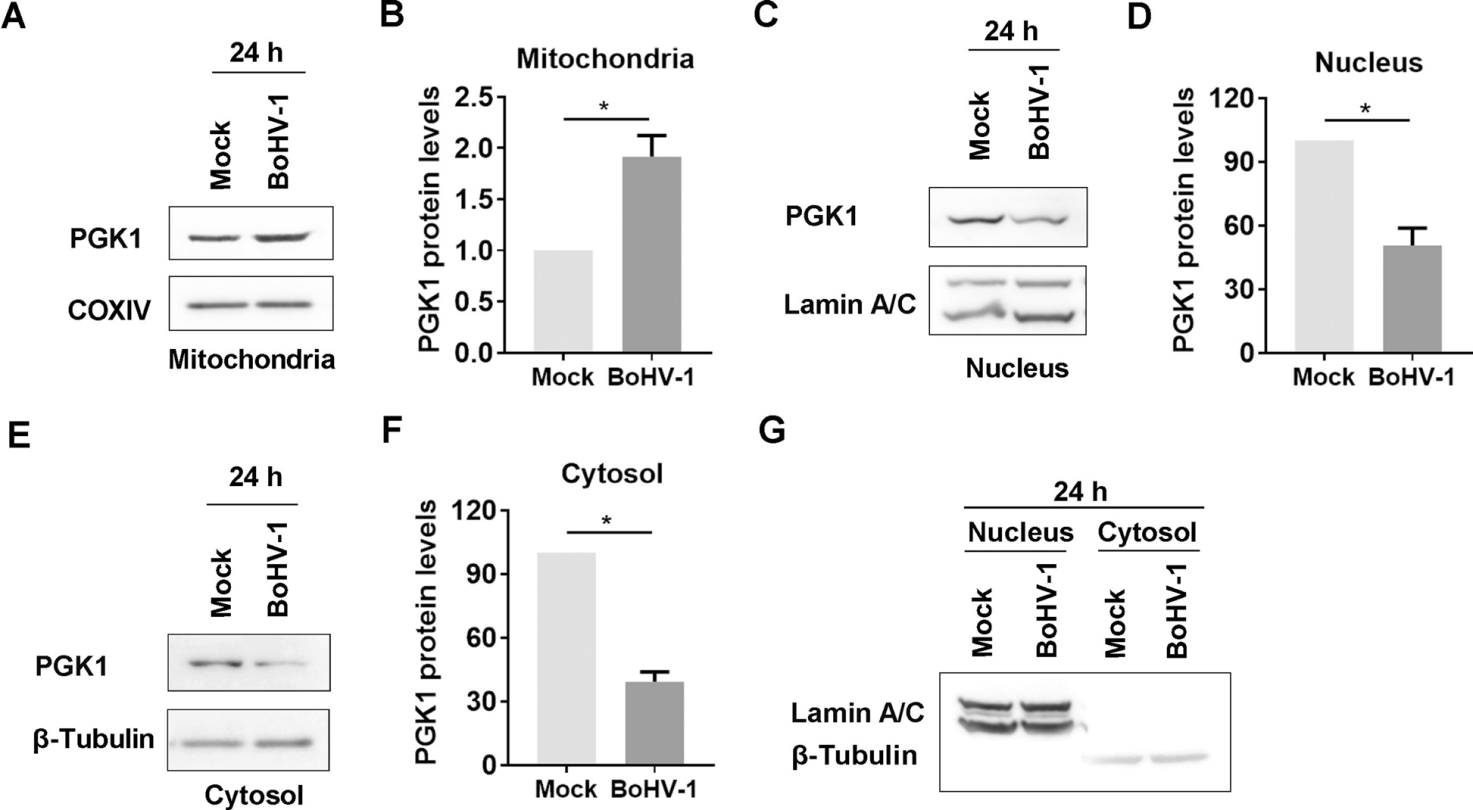
**BoHV-1 infection affects the accumulation of PGK1 proteins in distinct subcellular fractions.** **A**, **C**, **E** and **G** MDBK cells in 100 mm dishes were either mock-infected or infected with BoHV-1 (MOI = 1) for 24 h. The cells were collected for isolation of mitochondrial fractions using a commercial kit (Beyotime Biotechnology, cat# C3601) (**A**), as well as both nucleus and cytosol fractions via using a commercial nucleus isolation kit (Beyotime Biotechnology, cat# P0027) (**C**, **E** and **G**), following the manufacturer’s protocol. Then, they were subjected to PGK1 detection by western blotting. COXIV, LaminA/C, and β-Tubulin were probed as a protein loading control (**A**, **C** and **E**). **G** LaminA/C, a marker for nuclear protein, and β-tubulin, a marker for cytoplasmic protein, were detected in the cytosol and nucleus fractions, respectively, by western blot to determine if these fractions were contaminated by the counterparts. **B**, **D**, and **F** The band intensity was analysed using free software image J. The intensity of PGK1 was initially normalised to either LaminA/C or β-tubulin, and the fold change after infection was calculated by comparison to that of mock-infected controls, which were arbitrarily set as 1 or 100%. The results shown are means of three independent experiments, with error bars indicating standard deviations. Significance was assessed with Student’s *t*-test (* *p* < 0.05).

To investigate whether the accumulation of proteins in mitochondria is affected by viral infection at 24 h after infection, we purified mitochondrial proteins using a commercial kit (Beyotime Biotechnology, cat# C3601), and PGK1 was immunoprobed via western blotting. The results showed that viral infection led to an increased accumulation of PGK1 protein in the mitochondria (Figure 3A). Specifically, after viral infection, the levels of mitochondrial PGK1 protein rose nearly 1.92-fold compared to mock-infected cells (Figure 3B). This indicates that BoHV-1 productive infection enhances the accumulation of PGK1 protein in mitochondria at later stages.

To further investigate how PGK1 proteins were re-localised following virus infection, we purified fractions from the cytosol and nucleus of MDBK cells that were either mock-infected or virus-infected. This purification was performed using a commercial nucleus isolation kit (Beyotime Biotechnology, cat# P0027), and the samples were then subjected to western blot analysis.

We detected PGK1 protein in fractions of both the cytosol and nucleus. However, at 24 hpi, the levels of PGK1 protein in both fractions significantly decreased (Figures 3C and E). Specifically, the protein content was reduced to approximately 50.57% in the nucleus, while in the cytosol, it dropped to around 39.28% (Figures 3D and F). LaminA/C and β-tubulin, specific markers for nuclear and cytosol proteins, respectively, were not detected in the corresponding fractions (Figure 3G). This indicates no contamination between the fractions and supports our conclusion that viral infection results in the depletion of PGK1 protein in both fractions. These findings reinforce our hypothesis that PGK1 protein levels are reduced following viral infection.

These results indicate that differential viral infection may lead to the re-localisation of PGK1 proteins.

### PGK1 plays an important role in BoHV-1 productive infection

**Figure 4:**
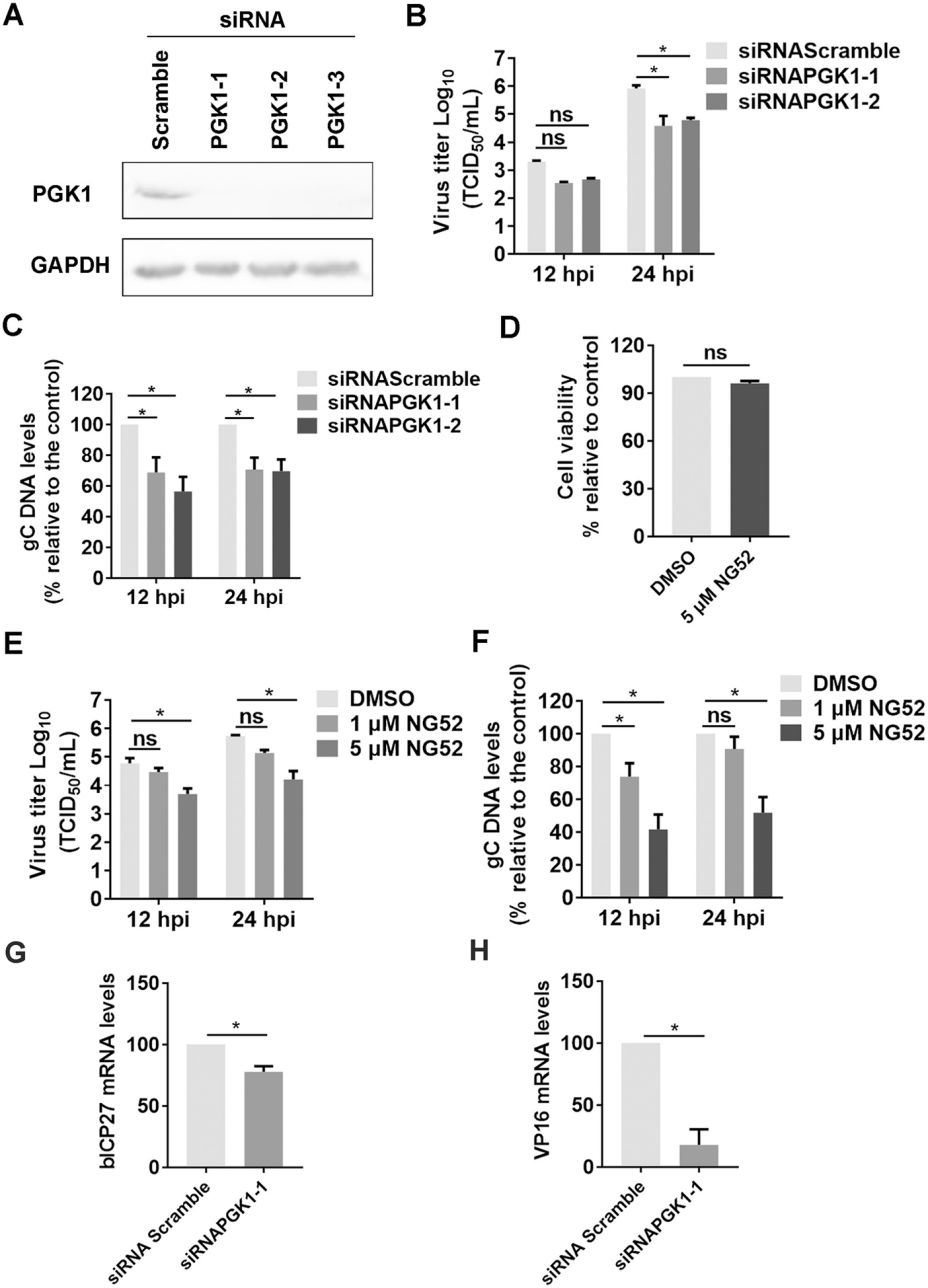
**PGK1 plays an essential role in BoHV-1 productive infection.** **A** MDBK cells in 6-well plates were transfected with either scrambled siRNA (200 pmol) or three individual siRNA targeting PGK1 (200 pmol), referred to as siRNAPGK1-1, siRNAPGK1-2, and siRNAPGK1-3, respectively. At 48 h post-transfection, PGK1 protein levels were detected via western blot. **B**, **C** MDBK cells in 6-well plates were transfected with 200 pmol of scrambled siRNA, siRNAPGK1-1, and siRNAPGK1-2, respectively. After transfection for 36 h, the cells were infected with BoHV-1 (MOI = 0.1) for one h. After three washes with PBS, the fresh medium was replaced. At 12 and 24 hpi virus yield in the cell cultures was measured, with results expressed as TCID_50_/mL (**B**), and levels of viral DNA were examined from intracellular content using qPCR with gC-specific primers (**C**). **D** The cytotoxicity of NG52 (5μM) in MDBK cells for 24 h was analysed by Trypan-blue exclusion test. **E** and **F** MDBK cells in 24-well plates pretreated with either DMSO control or NG52 at indicated concentrations were infected with BoHV-1 (MOI = 0.1) for one hour along with treatment-indicated chemicals. After three washing with PBS, fresh media containing DMSO control or NG52 were added for further incubation. At 12 and 24 hpi, the virus titers were measured, with the results expressed as TCID_50_/mL (**E**), and the intracellular content of virus genomic DNA was determined using relative qPCR with gC-specific primers (**F**). **G** and **H** MDBK cells in 6-well plates were transfected with 200 pmol of scrambled siRNA or siRNAPGK1-1. After transfection for 36 h, they were infected with BoHV-1 (MOI = 0.1) for 24 h. Total RNA was purified, and mRNA levels of bICP27 (**G**) and VP16 (**H**) were subsequently detected via qRT-PCR, respectively. The results shown are the means of three independent experiments, with error bars indicating standard deviations. Significance was assessed with Student’s *t*-test (* *p* < 0.05; ns = insignificant).

Since BoHV-1 productive infection affects the steady-state protein expression and subcellular localisation of PGK1, we investigated whether PGK1 plays a significant role in virus replication. We utilised three commercially available siRNAs—siRNAPGK1-1, siRNAPGK1-2, and siRNAPGK1-3—that can effectively knock down PGK1 protein expression in MDBK cells, as confirmed by western blotting (Figure 4A).

MDBK cells were transfected with either scrambled-siRNA or PGK1-specific siRNA (including siRNAPGK1-1 and siRNAPGK1-2) and subsequently infected with BoHV-1 at an MOI of 0.1. After 12 hpi, the virus titers decreased by approximately 0.76- and 0.64-log for siRNAPGK1-1 and siRNAPGK1-2, respectively, and by approximately 1.33- and 1.13-log at 24 hpi (Figure 4B).

We also assessed virus yields by detecting the intracellular viral genome using relative qPCR. Consistent with the viral titers, the amount of viral genome was reduced to about 68.88% and 56.42% at 12 hpi, and 70.67% and 69.67% at 24 hpi for siRNAPGK1-1 and siRNAPGK1-2, respectively (Figure 4C).

Thus, the knockdown of PGK1 expression using siRNA decreases viral productive infection, indicating that PGK1 signalling is required for the efficient replication of BoHV-1 in MDBK cells.

NG52 is a chemical inhibitor of PGK1 that targets explicitly and inhibits PGK1 kinase activity [36, 37]. We analysed the effect of NG52 on virus production in MDBK cells at concentrations ranging from 1 to 5 μM. At a concentration of 5 μM, NG52 did not exert significant toxic effects on MDBK cells (Figure 4D), as determined by the trypan blue exclusion test described by Fiorito et al. [38]. However, it significantly reduced virus titers by more than 1-log at 12 and 24 hpi, respectively (Figure 4E). After 12 and 24 h of infection, the copy number of the viral genome decreased by approximately 41.70% and 51.80%, respectively, with the application of 5 μM of NG52 (Figure 4F). In contrast, the lower concentration of 1 μM NG52 showed no effect on virus yield at either timepoint, as indicated by virus titers (Figures 4E and F). These results consistently suggest that NG52 inhibits virus-productive infection in a dose-dependent manner. Thus, we have demonstrated that PGK1 signalling is essential for virus replication using chemical inhibitors and siRNA-specific RNAs.

To better understand how PGK1 contributes to BoHV-1 productive infection, IFA was conducted to determine whether PGK1 co-localises with virion-associated proteins. However, co-localisation between PGK1 and the virion-associated proteins was not clearly observed (data not shown). This suggests that PGK1 may regulate viral replication in a manner that is independent of binding to these specific viral proteins. The impact of PGK1 knockdown on viral gene transcription—specifically bICP27 and VP16—was assessed using qRT-PCR. After the PGK1 protein was knocked down, the mRNA levels of bICP27 and VP16 were reduced to approximately 77.67% and 17.8%, respectively, 24 h after infection (Figures 4G and H).

These findings indicate that the knockdown of PGK1 may lead to decreased viral gene transcription, which could explain the reduced virus titers observed.

### β-catenin negatively regulates PGK1 protein expression

β-catenin is a cellular transcription factor that stimulates transcription of a panel of genes with either TCF/LEF-dependent or independent mechanisms [39, 40]. iCRT14 is a β-catenin-specific inhibitor, which inhibits β-catenin-dependent transcription by disrupting the interaction between β-catenin and TCF family members [41].

The effect of the β-catenin signalling pathway on PGK1 expression was initially assessed using the β-catenin-specific inhibitor iCRT14. Our results demonstrated that a concentration of 10 μM iCRT14 does not have toxic effects on MDBK cells and effectively inhibits β-catenin-dependent transcriptional activity [39, 42]. Consequently, MDBK cells were treated with iCRT14 at concentrations ranging from 0.1 to 10 µM for this study.

**Figure 5:**
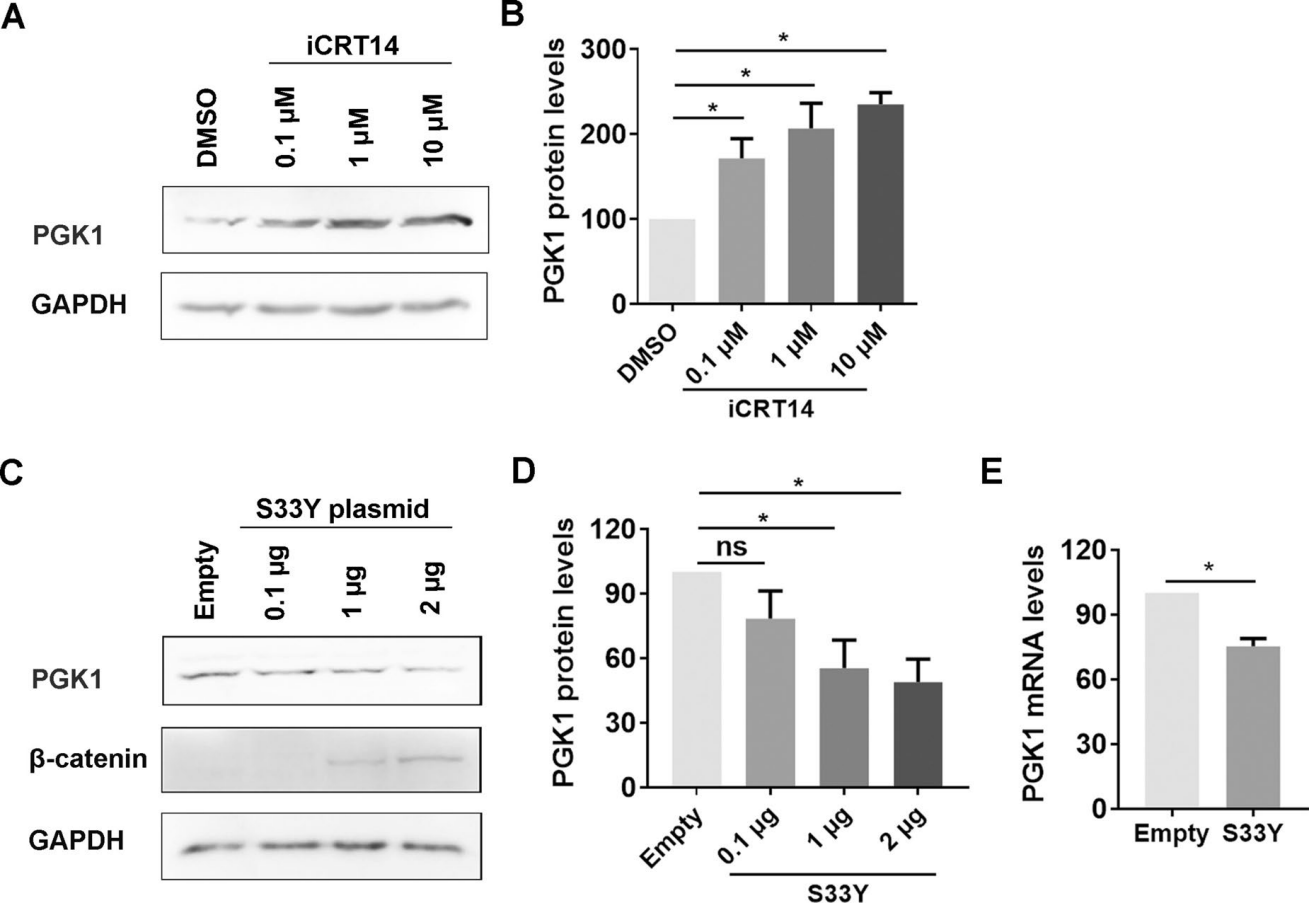
**β-catenin negatively regulates PGK1 protein expression.** **A** At indicated concentrations, MDBK cells in 6-well plates were treated with either DMSO control or β-catenin-specific inhibitor iCRT14. After treatment for 24 h, the cell lysates were prepared and subjected to western blot to detect PGK1 protein levels. **C** S33Y of 0.1, 1, and 2 μg, as well as an empty vector used as a control, were transfected into Neuro-2A cells in 6-well plates using lipofectamine 3000. After transfection for 48 h, cell lysates were prepared using RIPA lysis buffer and subjected to detection of both β-catenin and PGK1 proteins using western blot. **B**, **D** Band intensity was analyzed with free software Image J. The control was arbitrarily set as 100%. **E** Plasmid of either S33Y or empty vector of 1 μg was transfected into Neuro-2A cells in 6-well plates using lipofectamine 3000. After transfection for 48 h, total RNA was purified, and the mRNA levels of PGK1 were examined via using qRT-PCR. The control was arbitrarily set as 100%. The results shown are the means of three independent experiments, with error bars indicating standard deviations. Significance was assessed with Student’s *t*-test (* *p* < 0.05; ns = not significant).

We found that iCRT14 promoted PGK1 protein expression at all tested concentrations (Figure 5A). Following treatment with iCRT14 at concentrations of 0.1, 1, and 10 µM, the levels of β-catenin protein increased to approximately 1.71-, 2.07-, and 2.35-fold, respectively (Figure 5B).

When the plasmid expressing the constitutively active S33Yβ-catenin mutant was transfected into Neuro-2A cells, the expression levels of PGK1 protein decreased in a dose-dependent manner (Figure 5C). Compared to the control group transfected with a blank plasmid, PGK1 protein levels were reduced to approximately 55.37% and 48.87% following the transfection of 1 µg and 2 µg of the S33Y plasmid, respectively (Figure 5D). Additionally, after transfection of 1 µg of S33Y plasmid, PGK1 mRNA levels dropped to about 75.23% (Figure 5E). This suggests that the overexpression of activated β-catenin via plasmid transfection may inhibit PGK1 mRNA expression.

The data indicate that β-catenin signalling negatively affects the steady-state expression of PGK1 in MDBK cells.

### PGK1 positively regulates β-catenin expression, which is reversed following virus infection

**Figure 6:**
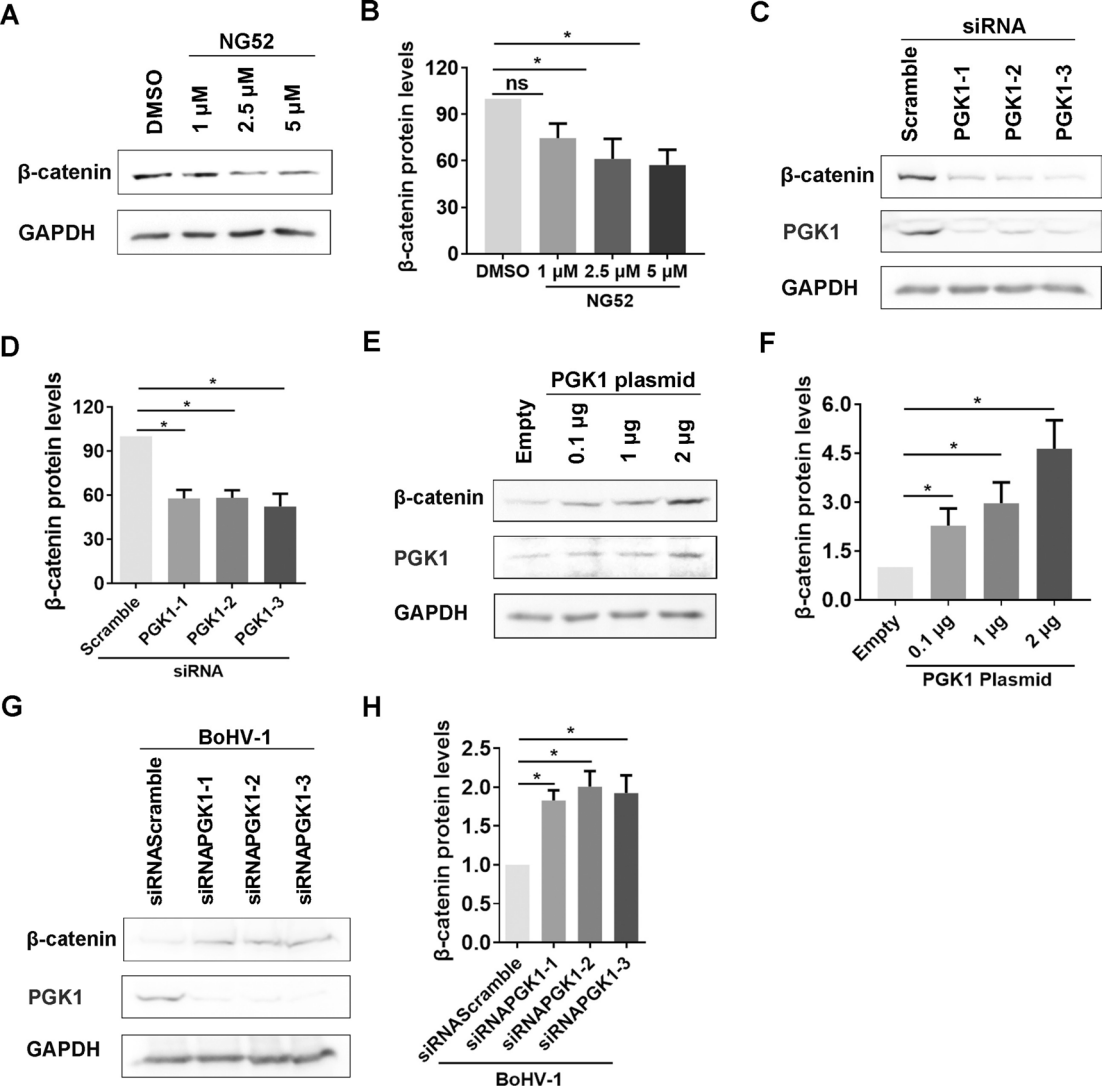
**PGK1 positively regulates β-catenin expression.** **A** MDBK cells in 6-well plates were treated with either DMSO control or NG52 at indicated concentrations for 24 h. The cell lysates were prepared and subjected to western blot to detect β-catenin protein levels. **C** MDBK cells in 6-well plates were transfected with 200 pmol of scrambled siRNA, siRNAPGK1-1, siRNAPGK1-2, and siRNAPGK1-3, respectively. At 48 h after transfection, β-catenin protein levels were detected by western blot. **E** PGK1 plasmid along with empty vector at the indicated dose were transfected into Neuro-2A cells in 6-well plates using lipofectamine 3000; after transfection for 48 h, the cells were either collected for the detection of β-catenin protein levels via western blot. **G** MDBK cells in 6-well plates were transfected with 200 pmol of scrambled siRNA or siRNAPGK1-1, siRNAPGK1-2, and siRNAPGK1-3, respectively. At 36 h after transfection, the cells were infected with BoHV-1 at an MOI of 0.1 for 24 h. Cell lysates were prepared with RIPA buffer and then protein levels of β-catenin were analysed using western blotting. GAPDH was probed as a loading control. **B**, **D**, **F**, and **H** Band intensity was analysed using the Image J software. The control was arbitrarily set as either 1 or 100%. The results shown are representations of three independent experiments, with error bars indicating standard deviations. Significance was assessed with Student’s *t*-test (*p < 0.05; ns = not significant).

Previous reports have suggested that β-catenin may be a downstream target of PGK1 [24]. A recent study indicated that disrupting the interaction between HSP90 and PGK1 stabilises β-catenin expression [25]. To characterise the effect of PGK1 on the β-catenin signalling pathway, we first assessed β-catenin protein expression in the presence of the PGK1-specific inhibitor NG52 using western blot analysis. We found that treatment with NG52 reduced β-catenin protein levels in a dose-dependent manner (Figure 6A). Specifically, compared to the DMSO control, β-catenin protein levels decreased to approximately 61.28% and 57.23% following treatment with 2.5 μM and 5 μM of NG52, respectively (Figure 6B). Additionally, β-catenin steady-state protein levels were significantly decreased by the knockdown of PGK1 expression using PGK1-specific siRNAs (Figure 6C). Compared to the scrambled-siRNA control, β-catenin protein levels declined to approximately 57.75%, 58.11%, and 52.28% with each of the three individual siRNA, respectively (Figure 6D).

Overexpression of the PGK1 protein through the transfection of PGK1 plasmid resulted in a significant increase in β-catenin expression in a dose-dependent manner (Figure 6E). Compared to the empty plasmid, the levels of β-catenin protein increased approximately 2.0-, 3.0-, and 4.6-fold with transfection of 0.1 μg, 1.0 μg, and 2.0 μg of PGK1 plasmid, respectively (Figure 6F). Therefore, the overexpression of the PGK1 protein increases β-catenin gene transcription.

**Figure 7:**
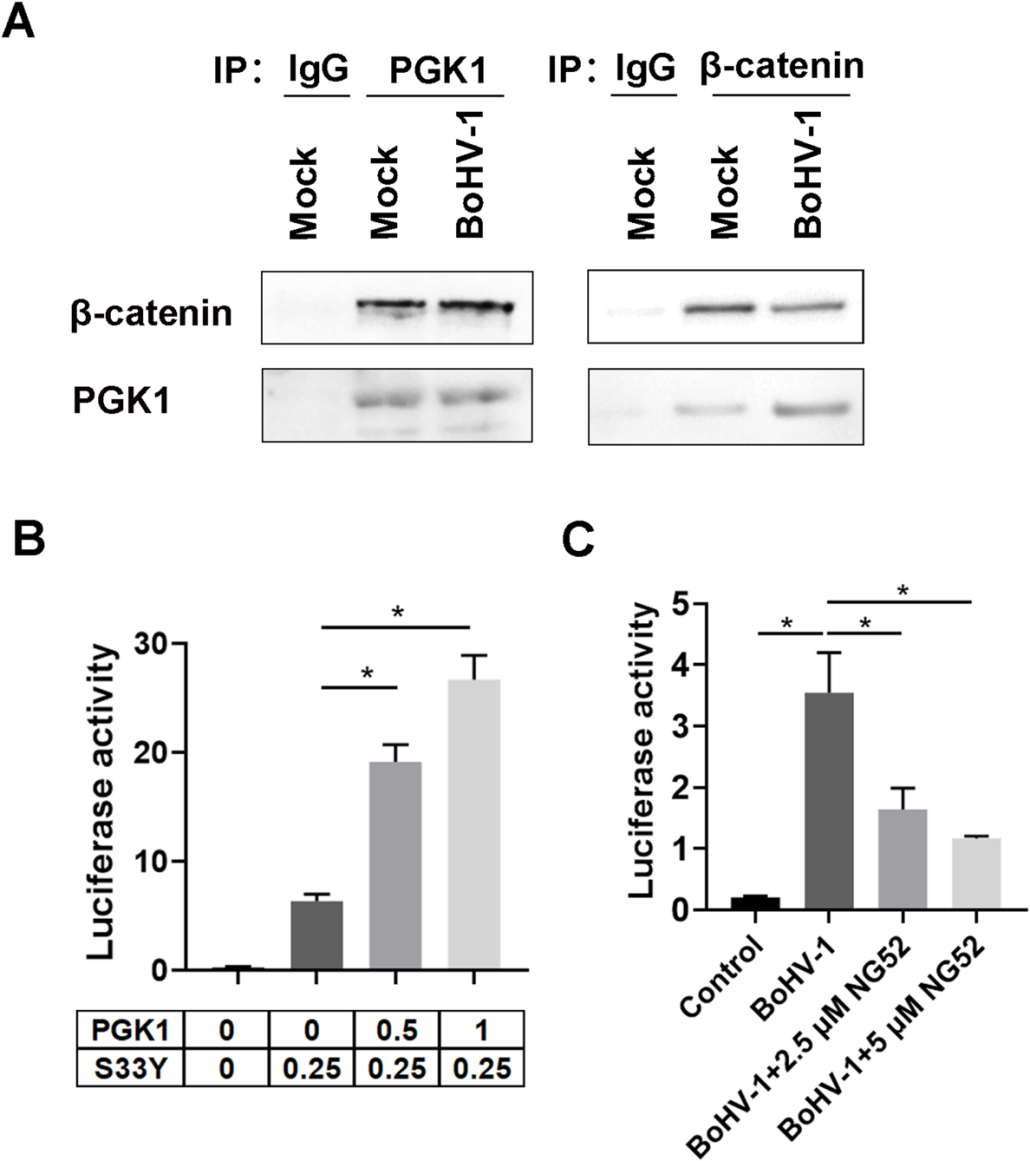
**PGK1 stimulates β-catenin-dependent transcription**. **A** MDBK cells in 60 mm dishes were mock infected or infected with BoHV-1(MOI = 0.1) for 24 h. Cell lysates were subjected to IP using antibodies against either PGK1 β-catenin or isotype IgG. Then both β-catenin and PGK1 were detected by western blot. The data shown are representative of three independent experiments. **B** Neuro-2A cells were co-transfected with 0.1 μg of the Super 8 × TOPFlash luciferase reporter construct, 0.01 μg of the Renilla reporter construct, and 0.25 μg of β-cateninS33Y mutant (S33Y) plasmid, together with increasing concentrations of a plasmid expressing PGK1 (0.5 or 1 μg) to examine the effect that PGK1 has on TCF promoter activity. At 48 h after transfection, dual luciferase assays were performed. **C** MDBK cells in 12-well plates (60% confluent) were transfected with 0.4 μg of the Super 8 × TOPFlash luciferase reporter construct and 0.05 μg of the Renilla reporter construct that was used as an internal control to allow normalisation of promoter activity. After transfection for 36 h, the cells were infected with BoHV-1 (MOI = 1) for 24 h along with treatment of either DMSO control or NG52 at the designated concentrations. Dual luciferase assays were performed 24 h after infection. The results shown are the average of three independent experiments, with error bars indicating standard deviations. Significance was assessed with Student’s *t*-test (*p < 0.05; ns, not significant).

Our study found that the PGK1 protein positively regulates the expression of β-catenin protein. We questioned whether the regulatory relationship holds true during BoHV-1 productive infection. Surprisingly, when we knocked down PGK1 levels protein before virus infection, we observed a significant increase in β-catenin expression in virus-infected cells (Figure 6G). The levels of β-catenin protein rose by approximately 2.0-fold when using three PGK1-specific siRNAs, respectively (Figure 7H). This indicates that the effect of PGK1 signalling on β-catenin expression is reversed during virus infection.

### PGK1 associates with β-catenin and stimulates β-catenin-dependent transcription

To further characterise the relationship between PGK1 and β-catenin, we investigated whether PGK1 associates with β-catenin in both mock-infected and virus-infected cells through immunoprecipitation (IP) studies. When we immunoprecipitated PGK1 from whole cell lysates, β-catenin was consistently detected in the immunoprecipitates (Figure 7A, left panel). Conversely, when we used a β-catenin antibody for the IP, we detected PGK1 in the immunoprecipitates (Figure 7A, right panel). The specific binding to PGK1 or β-catenin by the individual antibodies in these co-IP studies was confirmed using isotype IgG (Figure 7A). These findings suggest that PGK1 associates with β-catenin in both mock-infected and virus-infected cells.

The activated nuclear β-catenin binds to TCF family members, displacing the bound corepressors and recruiting transcriptional co-activators to the carboxy-terminal transactivation domain of β-catenin. This process stimulates the transcription of promoters that contain TCF-binding sites [43]. The plasmid Super 8 × TOPFlash contains 8 TCF binding sites upstream of a minimal promoter, which drives firefly luciferase reporter expression, thereby allowing accurate measurements of β-catenin-dependent transcriptional activity [44].

With this rationale, we tested whether PGK1 functions as a coactivator of β-catenin-dependent transcription. Neuro-2A cells were transfected with Super 8 × TOPFlash and increasing doses of PGK1, and promoter activity was measured 48 h after transfection. PGK1 stimulated β-catenin-dependent transcription by more than 3.01- and 4.20-fold with the transfection of 0.5 μg and 1 μg of PGK1 plasmid, respectively (Figure 7B). Neuro-2A cells were chosen for this study because they are easily transfected, and β-catenin is expressed at lower levels, making it difficult to detect in these cells [45]. Therefore, β-catenin may serve as a potential coactivator of β-catenin-dependent transcription.

It has been reported that BoHV-1 productive infection in bovine kidney cells stimulates β-catenin-dependent transcription, which in turn promotes viral replication [39]. In this study, we investigated whether PGK1 is involved in this activation process by assessing the promoter activity of Super 8 × TOPFlash in BoHV-1-infected MDBK cells exposed to either DMSO or chemical inhibitor NG52. Our results showed that β-catenin-dependent transcription levels decreased with NG52 treatment (Figure 7C), indicating that PGK1 may stimulate β-catenin-dependent transcription levels during BoHV-1 infection. Therefore, PGK1 appears to stimulate β-catenin-dependent transcription both in the presence and absence of BoHV-1 infection, potentially promoting BoHV-1 replication through the activation of β-catenin-dependent transcriptional activity.

### PGK1 signalling promotes nuclear translocation of p-β-catenin (S552) following BoHV-1 productive infection

Nuclear localisation of β-catenin is essential for its transcriptional activation following stimulation [46]. Notably, phosphorylation of β-catenin at Ser552 [p-β-catenin (S552)] enhances its transcriptional activity [47]. Additionally, infection with BoHV-1 increases the phosphorylation of β-catenin at S552 and enhances the nucleus accumulation of p-β-catenin (S552) [42].

**Figure 8:**
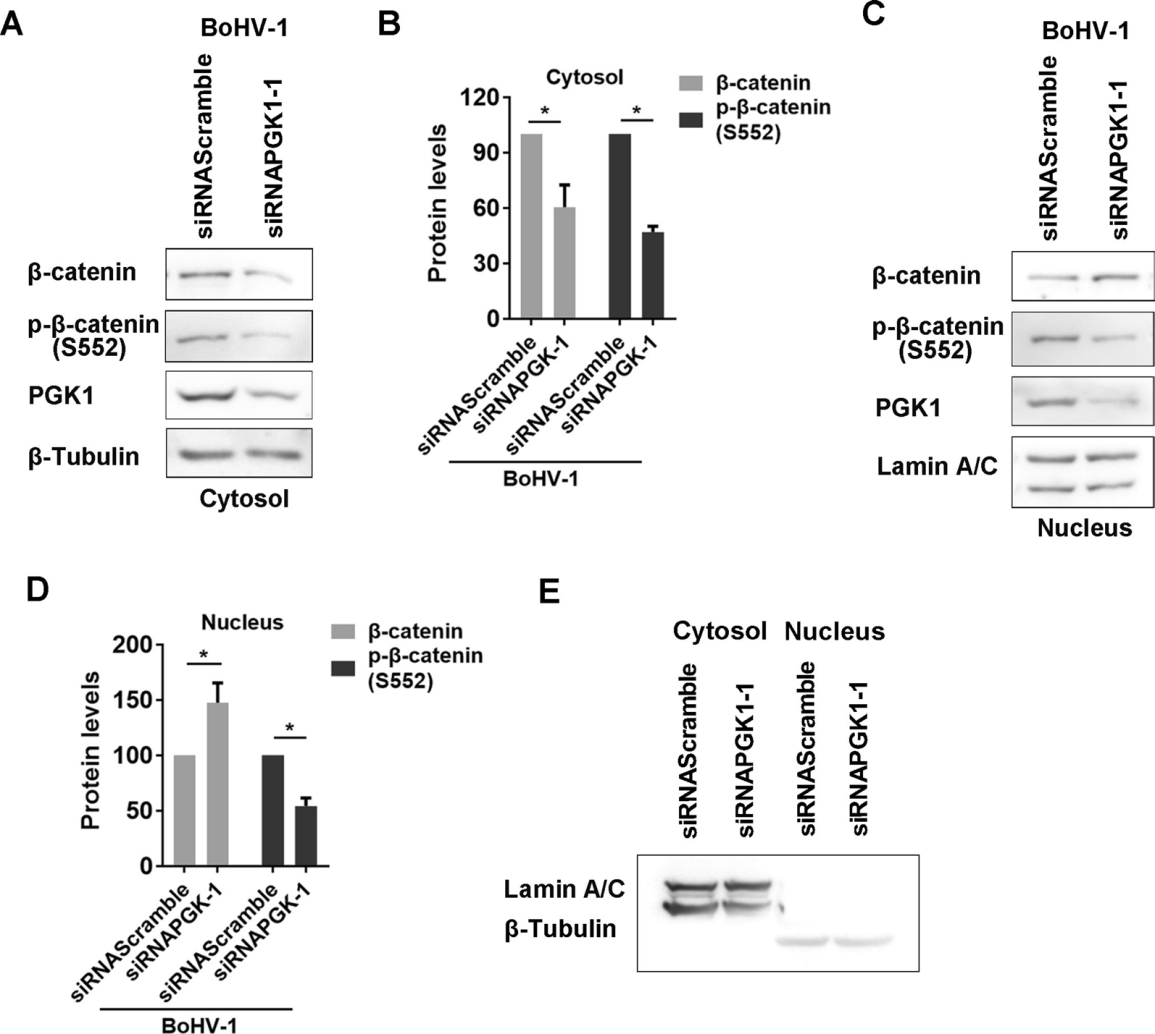
**PGK1 signaling promotes nucleus translocation of activated p-β-catenin (S552) stimulated by BoHV-1 productive infection**. **A** and **C** MDBK cells in 6-well plates were transfected with 200 pmol of either scrambled siRNA, or siRNAPGK1-1, respectively. After transfection for 36 h, the cells were infected with BoHV-1 (MOI = 1) for 24 h. Then the cells were collected to isolate both nucleus and cytosol fractions via a commercial kit (Beyotime Biotechnology, cat# P0027), following the manufacturer’s protocol. Protein levels of both β-catenin and p-β-catenin(S552) were detected from individual fractions by western blotting, respectively. **E** LaminA/C, a marker for nuclear protein, and β-tubulin, a marker for cytoplasmic protein, were detected in the fractions of both cytosol and nucleus using western blot. **B** and **D** Band intensity was analysed with software Image J. The control was arbitrarily set as 100%. The results shown are the means of three independent experiments, with error bars indicating standard deviations. Significance was assessed with Student’s *t*-test (*p < 0.05).

In this study, we examined whether PGK1 influenced the nuclear accumulation of p-β-catenin (S552) during viral infection. To investigate this, we isolated subcellular fractions from the cytosol and nucleus in virus-infected MDKB cells, both with and without PGK1 knockdown, using siRNA. The results showed that protein levels of both β-catenin and p-β-catenin (S552) in the cytosol fractions decreased to approximately 60.35% and 46.92%, respectively, following the PGK1 knockdown (Figures 8A and B).

Interestingly, PGK1 knockdown by siRNA led to a slight increase in the accumulation of β-catenin but resulted in a reduction of p-β-catenin (S552) in the nuclear fractions during virL infection (Figure 8C). Compared to the scrambled- siRNA, nuclear p-β-catenin (S552) protein levels dropped to approximately 54.05% after PGK1 knockdown (Figure 8D).

LaminA/C, a marker of nucleus proteins, was scarcely detected in the nuclear fractions (Figure 8E), indicating that cytosol proteins did not contaminate nuclear fractions. This validates our findings that the knockdown of PGK1 protein decreases the accumulation of p-β-catenin (S552) in the nucleus during BoHV-1 infection.

PGK1 may partially influence β-catenin activity by promoting the nuclear accumulation of p-β-catenin (S552) in virus-infected cells. This supports findings that inhibiting PGK1 chemically with NG52 decreases β-catenin-dependent transcriptional activity.

## Discussion

The role of PGK1 signalling has been documented in only two viruses: Senecavirus A (SVA) and Tomato bushy stunt virus (TBSV). Both viruses utilise PGK1 signalling for efficient infection [8, 15]. TBSV, a plant virus, co-opts PGK1 into the viral replication compartment to facilitate virus replication [15]. On the other hand, SVA, which infects swine and causes disease, increases PGK1 protein expression in PK-15 cells, promoting virus replication [8].

Interestingly, we found that BoHV-1 infection decreases PGK1 protein expression while promoting virus replication (Figure 4). Despite these viruses having distinct genomic types (RNA vs. DNA), naturally infecting distinct host species (mammals vs. plants), and causing different effects on PGK1 protein expression (increased vs. decreased), they all rely on PGK1 signalling for efficient replication.

Numerous studies suggest that PGK1 signalling may play a significant role in the pathogenicity of various diseases. For instance, the chemical inhibitor CBR-470-1, which blocks PGK1 signalling, has been shown to protect SH-SY5Y neuroblastoma cells from neurotoxin MPP+ (1-methyl-4-phenylpyridinium ion)-induced cytotoxicity. This protective effect is dependent on the activation of the anti-oxidant signalling Nrf2 [48]. Similarly, the chemical inhibition of PGK1 signalling by evodiamine can prevent traumatic brain injury (TBI) by reducing oxidative stress through the activation of Nrf2 signalling [49].

Moreover, the silencing of PGK1 has been found to decrease the inflammatory response triggered by oxygen–glucose deprivation/reoxygenation (OGD/R) in rat models of ischemic brain injury [50]. Together, these findings indicate that inhibiting PGK1 signalling through various methods may either mitigate disease development or promote recovery from these conditions in vivo, partially by activating Nrf2 signalling, which helps reduce oxidative stress.

On the other hand, increased mitochondrial accumulation of PGK1 has been linked to neuronal death after OGD/R injury, as it inhibits mitochondrial oxidative phosphorylation (OXPHOS) and stimulates glycolysis [35, 51, 52]. This suggests that mitochondrial PGK1 may have harmful effects on tissue damage.

Therefore, intervening in the PGK1 signalling pathway could offer a promising therapeutic strategy for a range of diseases. Additionally, it has been noted that BoHV-1 productive infection disrupts Nrf2 signalling, exacerbates oxidative stress [19, 53], and increases the accumulation of PGK1 in mitochondria (Figure 3A and B). This indicates that PGK1 signalling may be involved in the pathogenesis of diseases caused by BoHV-1 infection, providing a new avenue for developing therapeutic medications to treat virus-related diseases.

We discovered that activated β-catenin may act as a repressor of PGK1 expression. This is indicated by the observation that overexpression of the activated S33Yβ-catenin mutant led to a decrease in PGK1 protein levels. At the same time, chemical inhibition of β-catenin activity increased PGK1 levels (Figure 5). Furthermore, PGK1 signalling was found to regulate β-catenin expression positively and stimulated β-catenin-dependent transcription in uninfected cells as demonstrated by the transfection of either siRNA or PGK1 plasmid (Figures 6A–F).

These findings suggest a reciprocal regulation loop between PGK1 and β-catenin protein expression, with β-catenin signalling negatively regulating PGK1 expression and PGK1 signalling positively regulated β-catenin expression. However, BoHV-1 productive infection reversed the effects that PGK1 signalling had on β-catenin expression, as shown in mock-infected cells. Interestingly, PGK1 signalling stimulates β-catenin-dependent transcriptional activity both in the absence and presence of viral infection, as verified by luciferase assay (Figures 7B and C).

Further investigation revealed that the knockdown of PGK1 protein expression using siRNA significantly reduced the nuclear accumulation of p-β-catenin (S552) protein (Figure 8B and C). Given that the nuclear localisation and phosphorylation of β-catenin at Ser552 are prerequisites for its transcriptional activation [46, 47], the observed decrease in nuclear p-β-catenin (S552) protein levels due to PGK1 knockdown supports our findings that the PGK1-specific inhibitor NG52 inhibits β-catenin-dependent transcriptional activity stimulated by virus infection (Figure 8C).

Notably, several host factors, including axin, adenomatous polyposis gene (APC), glycogen synthase kinase 3β (GSK3β), and CKI [44], have been implicated in regulating β-catenin activity. Here, we provide the first evidence that PGK1 may also play a role as a host factor in regulating β-catenin activation.

We have previously reported that BoHV-1 productive infection stimulates β-catenin-dependent transcription and increases protein levels of activated p-β-catenin (S552), which is necessary for efficient replication [39, 42]. In this study, we reveal for the first time that PGK1 may regulate β-catenin activity and demonstrate a reciprocal regulation loop between PGK1 and β-catenin signalling pathways. Specifically, β-catenin signalling suppresses PGK1 expression, while PGK1 signalling stimulates β-catenin protein expression and β-catenin-dependent transcription in mock-infected cells. Additionally, PGK1 stimulates β-catenin-dependent transcriptional activity in virus-infected cells, partly by stimulating the nuclear accumulation of p-β-catenin (S552). This finding further clarifies how β-catenin signalling pathways stimulate BoHV-1 productive infection for efficient replication. Moreover, we have provided evidence that PGK1 is a potential target for developing novel therapeutic reagents against diseases related to BoHV-1.

## Data Availability

The datasets used and/or analysed during the current study are available from the corresponding author upon reasonable request.
